# Supplementary material for: The TAS1R2 G-protein-coupled receptor is an ambient glucose sensor in skeletal muscle that regulates NAD homeostasis and mitochondrial capacity
Source: Nat Commun. 2024 Jun 8;15:4915. doi: 10.1038/s41467-024-49100-8 (PMC11162498; doi:10.1038/s41467-024-49100-8)
Supplement: Supplementary file 1 — Supplementary Information [file 41467_2024_49100_MOESM1_ESM.pdf]

Supplementary figures

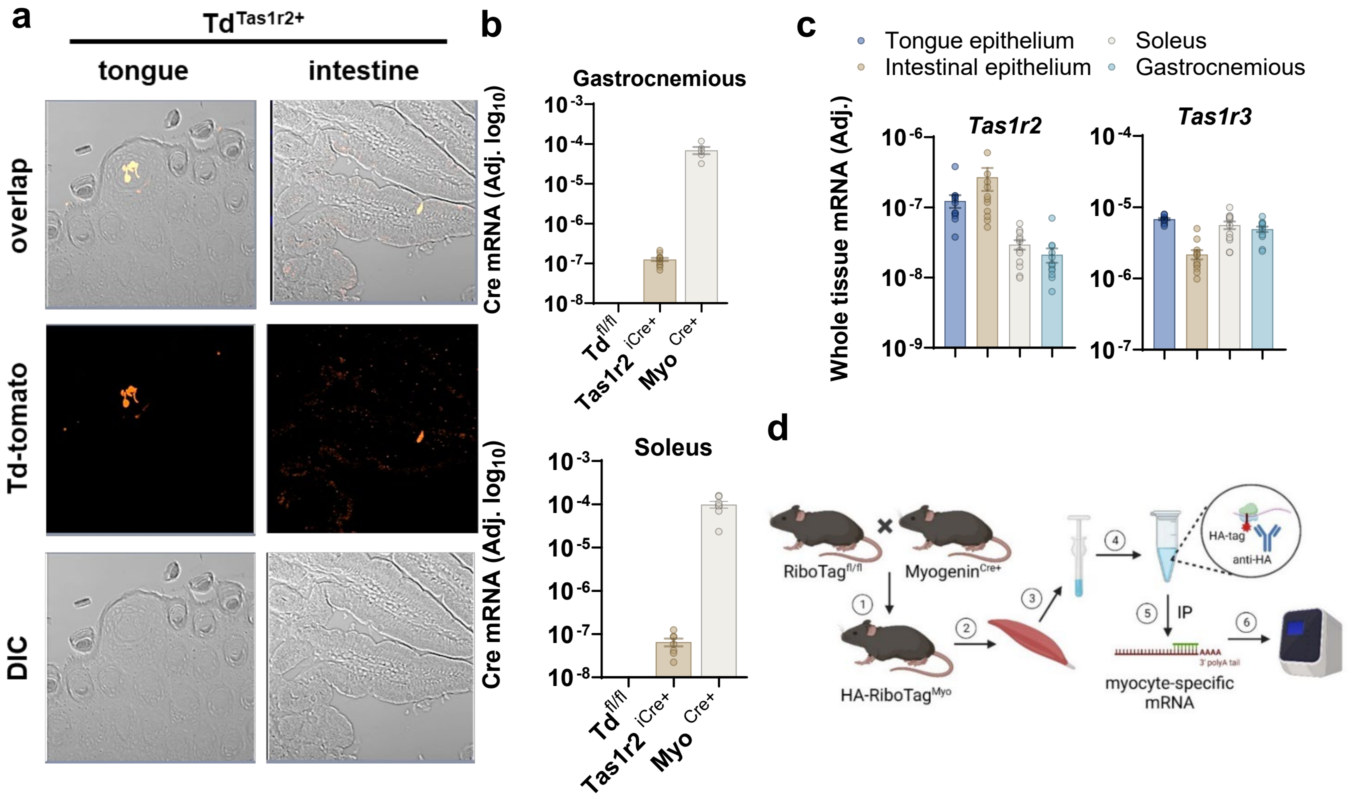

Supplementary Fig.1. The *Tas1r2* and *Tas1r3* sweet taste GPCRs are expressed in skeletal muscle fibers.
(a) Immunofluorescence of TdTomato in the tongue and intestine of Tas1r2-Cre:TdTomato-fl/fl mice (TdTas1r2+) showing Tas1r2 expressing cells in corresponding tissues. **(b)** Active Cre expression in gastrocnemius and soleus muscles of Tas1r2-Cre+ mice. Td-fl/fl muscles were used as a negative control and Myo-Cre+ muscles were used as a positive control (Gastrocnemious Td-fl/fl n=6; Tas1r2-Cre+ n=13; Myo-Cre+ n=5; Soleus Td-fl/fl n=6; Tas1r2-Cre+ n=7; Myo-Cre+ n=7). Data is presented as mean ± SEM. **(c)** Comparison of *Tas1r2* and *Tas1r3* expression in skeletal muscles (i.e., soleus and gastrocnemius) and reference tissues such as epithelia of the tongue and intestine (n=12 for all groups). Data is presented as mean ± SEM. **(d)** Description of mRNA isolation from Myogenin-Cre:HA-RiboTag-fl/fl muscles (see Methods). 1. HA-RiboTag mice (Rpl22fl/fl-HA) were obtained from the Jackson Lab (Jax# 029977) and crossed with Myogenin-Cre mice to generate a myocyte HA-RiboTag reporter (HA-RiboTagMyo). 2. Muscle samples were harvested and frozen. 3. Frozen samples were pulverized and homogenized. 4. Supernatant was incubated with anti-hemagglutinin (anti-HA) antibody. 5. mRNA was immunoprecipitated (IP) and extracted. 6 mRNA was further processed for assessing myocyte-specific gene expression. Panel (d) was created with BioRender.com released under a Creative Commons Attribution-NonCommercial-NoDerivs 4.0 International license. Source data are provided as a Source Data file.

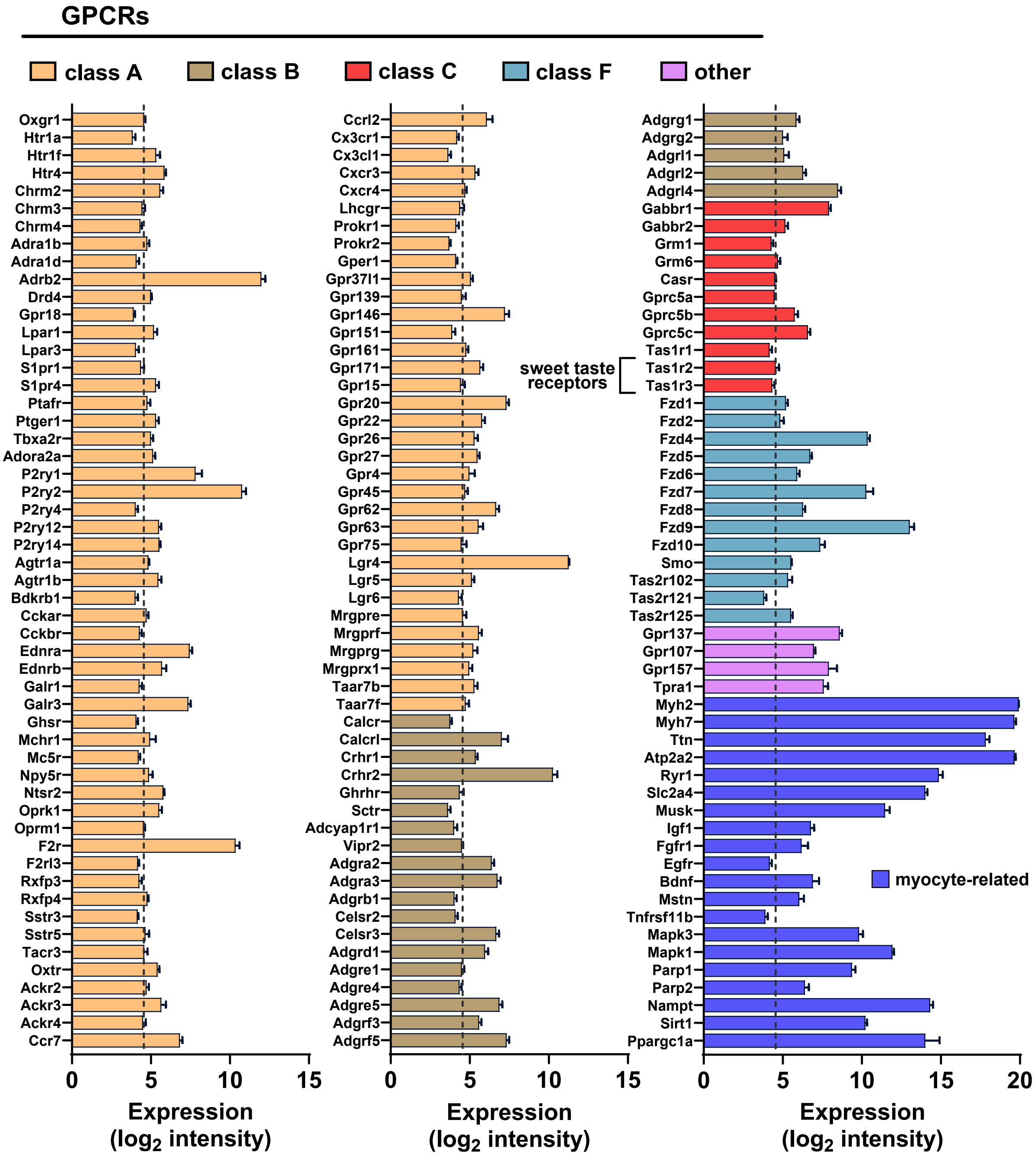

Supplementary Fig.2. GPCRs expression in skeletal myofibers (myocytes).
GPCR expression in skeletal myofibers from HA-RiboTagMyo mice (n=5) using transcriptomics analysis. GPCRs were grouped according to their class (A, B, C, F, or other). Odorant receptors are not shown. Expression of select myocyte-related genes is included as reference. Dotted line shows average Tas1r2 expression level. Data is presented as mean ± SEM. Source data are provided as a Source Data file.

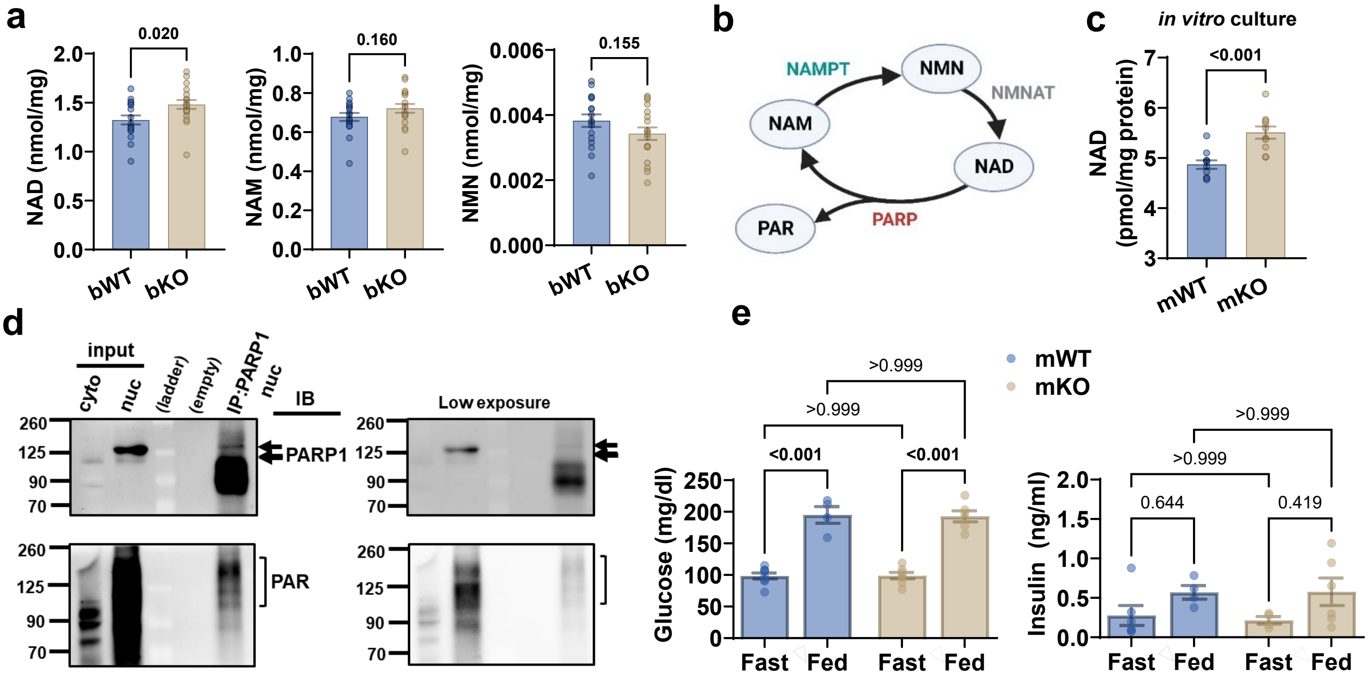

Supplementary Fig.3. ***TAS1R2-mediated glucose sensing regulates NAD levels coupled to PARP activity***.
(a) Quantitative targeted nucleotide analysis (NAD, NAM, and NMN) in bWT and bKO muscles using LC/MS (bWT n=17; bKO n=18). Data is presented as mean ± SEM. Two-sided t-tests. **(b)** Diagram shows the salvage pathway of NAD biosynthesis. **(c)** NAD concentration in acidic extracts of differentiated primary myocyte cultures from mWT and mKO muscles (n=10 for each group). Data is presented as mean ± SEM. Two-sided t-test. **(d)** Immunoblotting (IB) of PARP1 and PAR in mWT nuclear muscle lysates immunoprecipitated (IP) with PARP1 antibody. PAR between 115-180KDa is specific to PARP1 activity. Two exposure times are shown for clarity. **(e)** Plasma glucose and insulin concentrations of mWT and mKO mice subjected to 24h fast (Fast) followed by 2h feeding (Fed) (glucose: fasted mWT, n=8; fasted mKO, n=7; fed mWT, n=4; fed mKO, n=6; insulin: fasted mWT, n=6, fasted mKO, n=4; fed mWT, n=4; fed mKO, n=6). Two-way ANOVA, Sidak post-hoc effect. Panel (b) was created with BioRender.com released under a Creative Commons Attribution-NonCommercial-NoDerivs 4.0 International license. Source data are provided as a Source Data file. Blots were repeated twice with similar results.

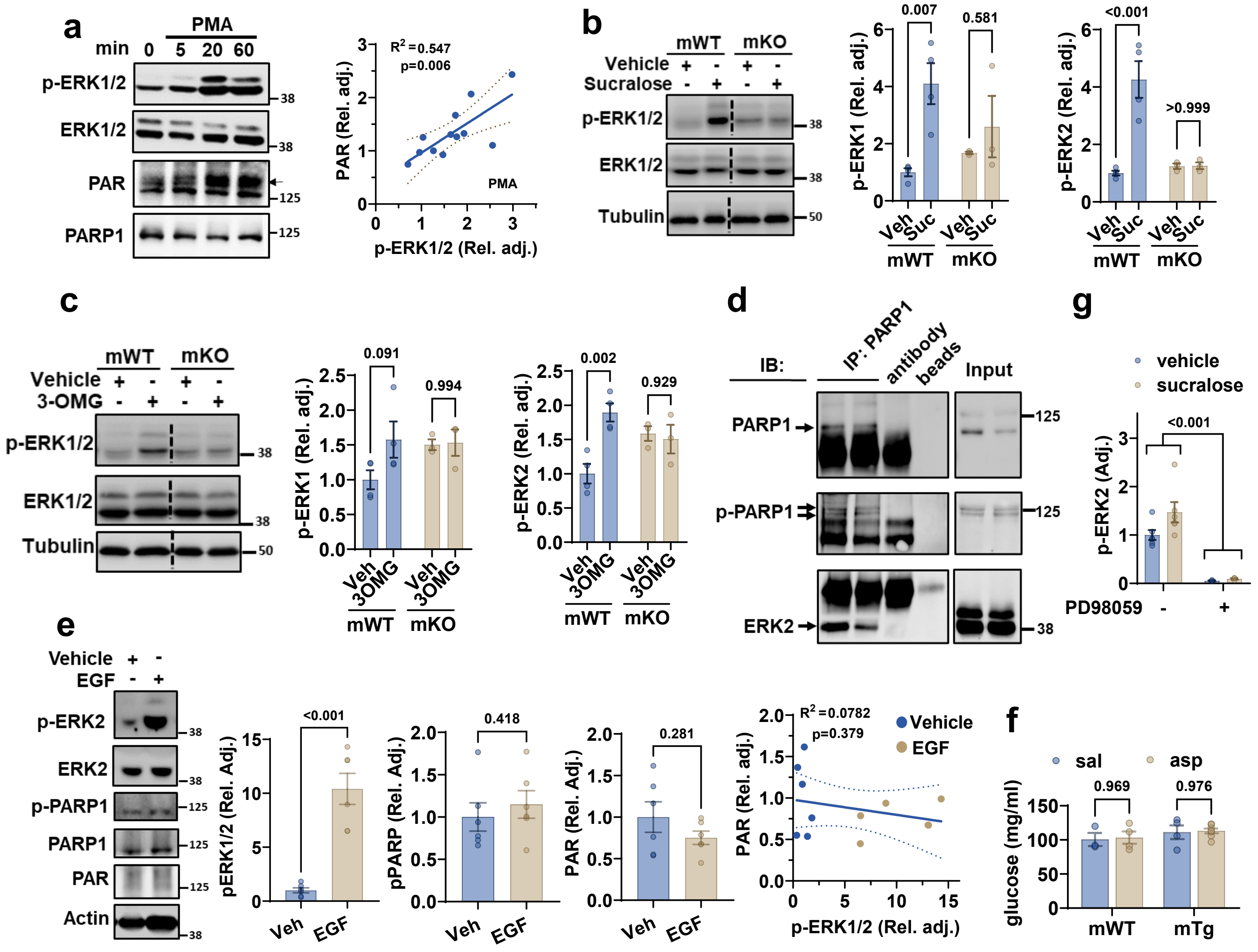

Supplementary Fig.4. TAS1R2 activates the ERK2-PARP1 axis in skeletal muscle.
(a) Immunoblotting of p-ERK1/2, PAR, and PARP1 in C2C12 cells following treatment with the ERK1/2 activator, PMA (left). Simple linear regression of p-ERK1/2 with PAR in PMA-treated C2C12 cells (right) (n=12). F-test, slope p-value. **(b)** Immunoblotting and quantitation of p-ERK1/2 in response to intramuscular injection of sucralose or vehicle in mWT and mKO muscles (mWT vehicle n=4; mWT sucralose, n=4; mKO vehicle, n=3; mKO sucralose, n=3). Data is presented as mean ± SEM. Two-way ANOVA, Sidak post-hoc effect. **(c)** Immunoblotting and quantitation of p-ERK1/2 in response to intramuscular injection of 3-OMG or vehicle in mWT and mKO muscles. (mWT vehicle n=4; mWT 3-OMG, n=4; mKO vehicle, n=3; mKO 3-OMG, n=3). Data is presented as mean ± SEM. Two-way ANOVA, Sidak post-hoc effect. **(d)** Immunoblotting (IB) of p-PARP and ERK2 in mWT nuclear muscle lysates immunoprecipitated (IP) with PARP1. **(e)** Immunoblotting and quantitation of p-ERK, p-PARP1, and PAR in response to intramuscular injection of EGF or vehicle in WT muscles (n=6 for all groups). Data is presented as mean ± SEM. Two-sided t-tests. Simple linear regression of p-ERK1/2 with PAR in Vehicle- or EGF-treated muscles (n=12). F-test, slope p-value. **(f)** Average (3h) plasma glucose of 24h fasted mWT and mKO mice treated with i.p. saline (sal) or aspartame (asp) (mWT saline, n=3; mWT aspartame, n=4; mTg saline, n=4; mTg aspartame, n=7). Data is presented as mean ± SEM. Two-way ANOVA, Sidak post-hoc effect. **(g)** Quantitation of p-ERK2 in C2C12 cells following treatment with sucralose with or without PD98059 (vehicle control, n=6; vehicle sucralose, n=6; PD98059 control, n=3; PD98059 sucralose, n=3). Data is presented as mean ± SEM. Two-way ANOVA, inhibitor treatment effect (brackets) p<0.001. Source data are provided as a Source Data file. Blots were repeated twice with similar results.

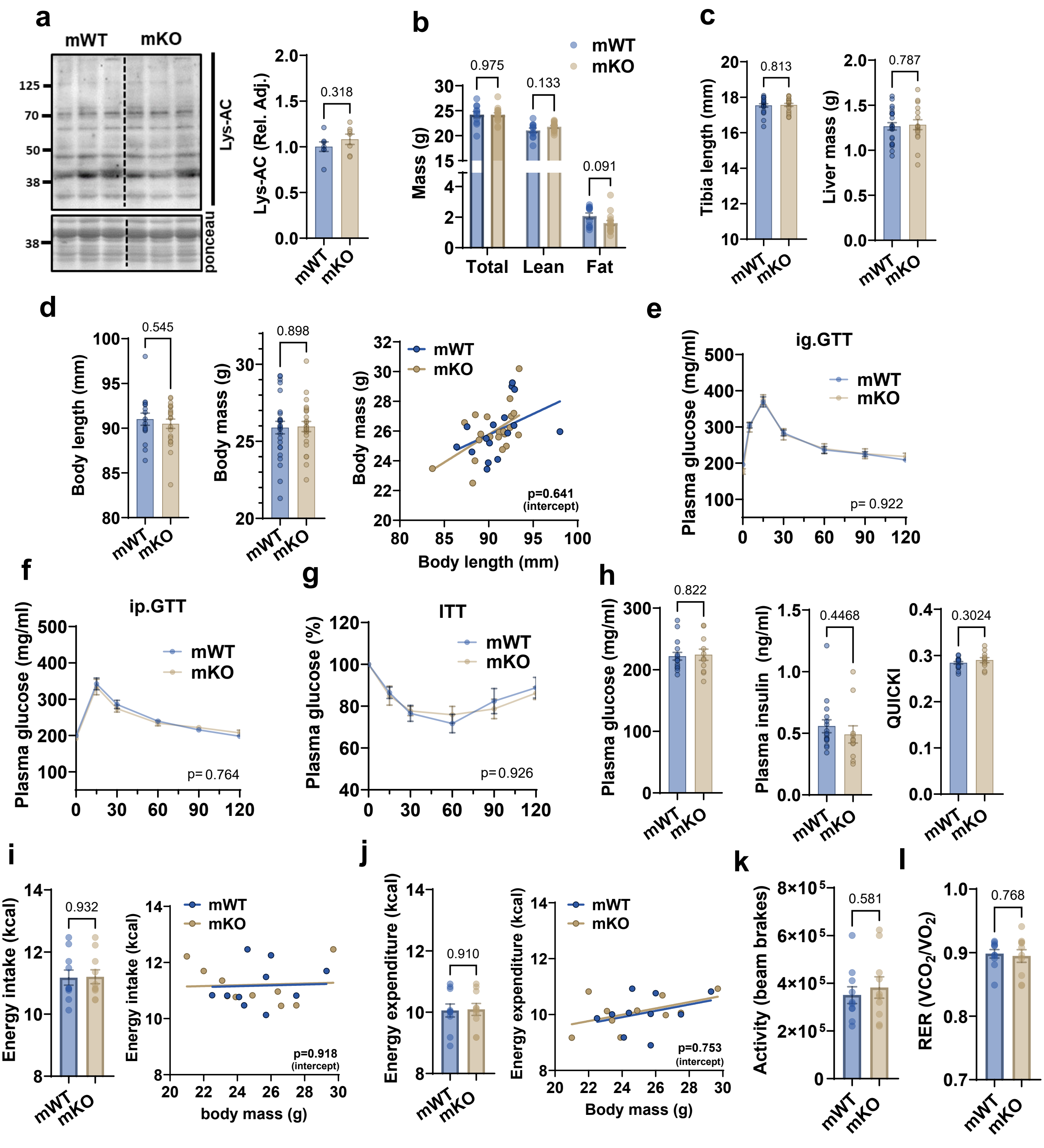

Supplementary Fig.5. TAS1R2 deficiency in skeletal muscle does not affect growth and glucose homeostasis. **(a)** Quantitation and immunoblotting of total cell acetylation (Lys-Ac) in mWT and mKO muscles (n=7 for each group). Ponceau is loading control. Data is presented as mean ± SEM. Two-sided t-test. **(b)** Total body, lean and fat mass in mWT and mKO mice (mWT, n=11; mKO, n=15). Data is presented as mean ± SEM. Two-sided t-tests. **(c)** Tibia length and liver mass of mWT and mKO mice (tibia mWT, n=21; tibia mKO, n=24; liver mWT, n=22; liver mKO, n=16). Data is presented as mean ± SEM. Two-sided t-tests.**(d)** Absolute values and simple linear regression of body length and body mass in mWT and mKO mice (length mWT n=16, length mKO, n=21; mass mWT n=25, mass mKO, n=24; regression n=37). Data is presented as mean ± SEM. Two-sided t-tests. Regression F-test, intercept p-value. **(e)** Intragastric (i.g.) glucose tolerance test (GTT), **(f)** intraperitoneal (i.p.GTT), and **(g)** insulin tolerance test (ITT) in mWT and mKO mice (i.g.GTT n=9 for each group; i.p.GTT mWT, n=10; i.p.GTT mKO, n=11; ITT, n=10 for each group). Data is presented as mean ± SEM. Two-way ANOVA, p-value main genotype effect. **(h)** Plasma glucose, insulin and quantitative insulin-sensitivity check index (QUICKI) in adlib fed mWT and mKO mice (mWT, n=16; mKO, n=11). Data is presented as mean ± SEM. Two-sided t-tests. Daily average **(i)** energy intake, **(j)** energy expenditure, **(k)** activity, and **(l)** respiratory exchange ratio (RER) in free-living mWT and mKO mice (n=10 for each group, regressions n=20). Data is presented as mean ± SEM. Two-sided t-tests. Regression F-tests, intercept p-value. Source data are provided as a Source Data file. Blots were repeated twice with similar results.

Blot scans

2b

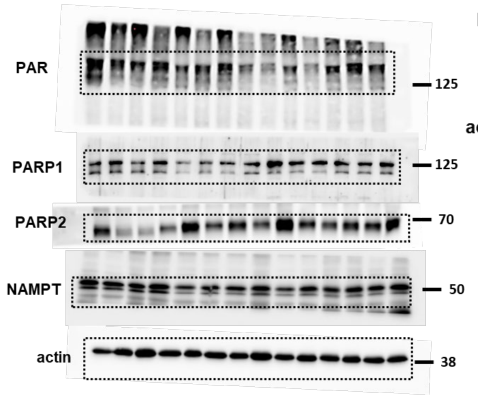

2c

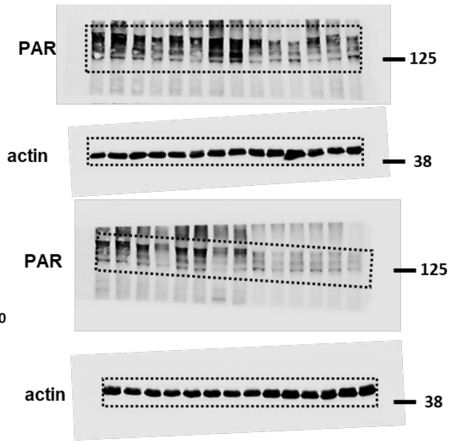

2f

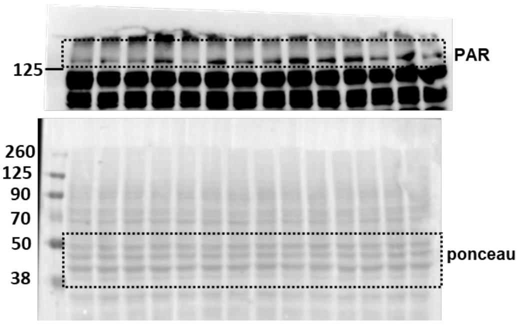

2i

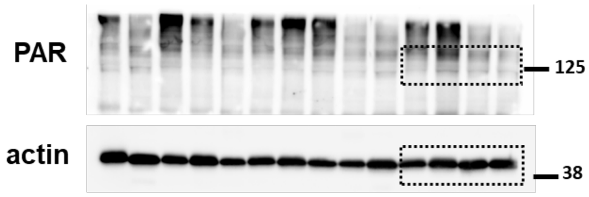

3a

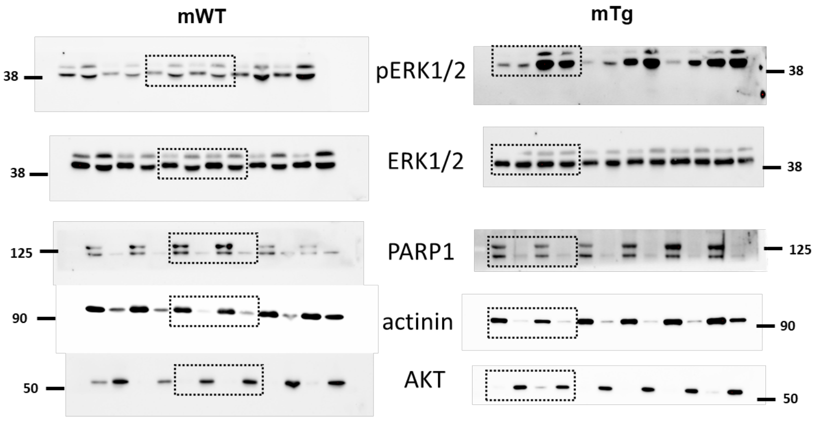

3b

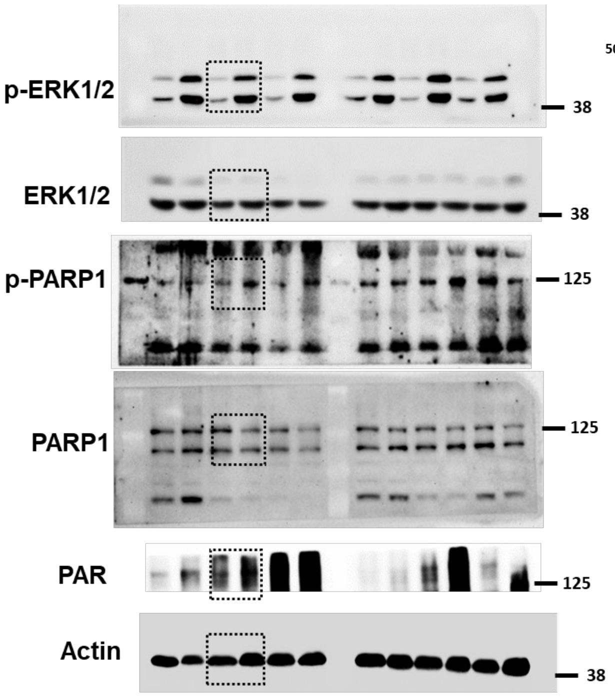

3d

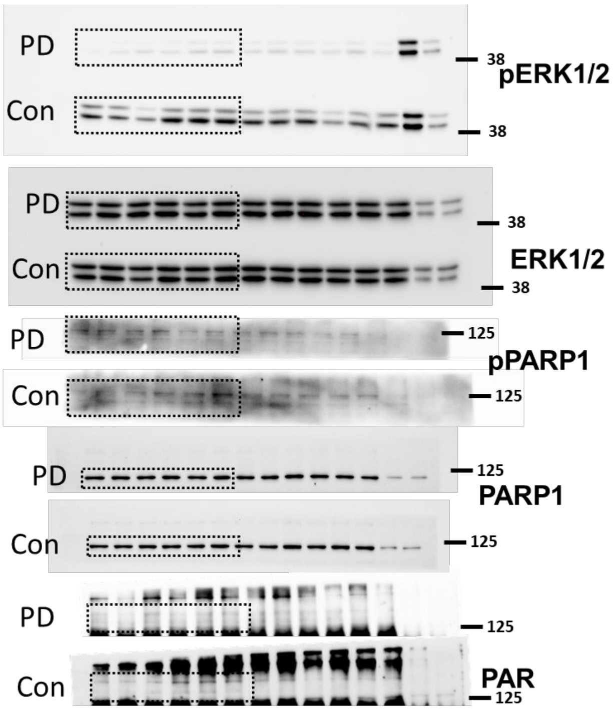

4a

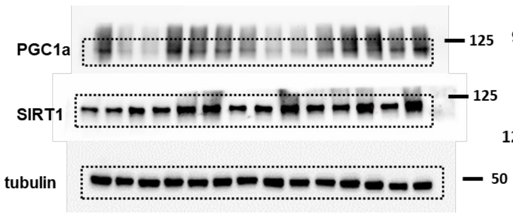

4b

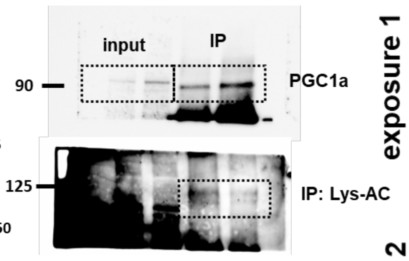

4d

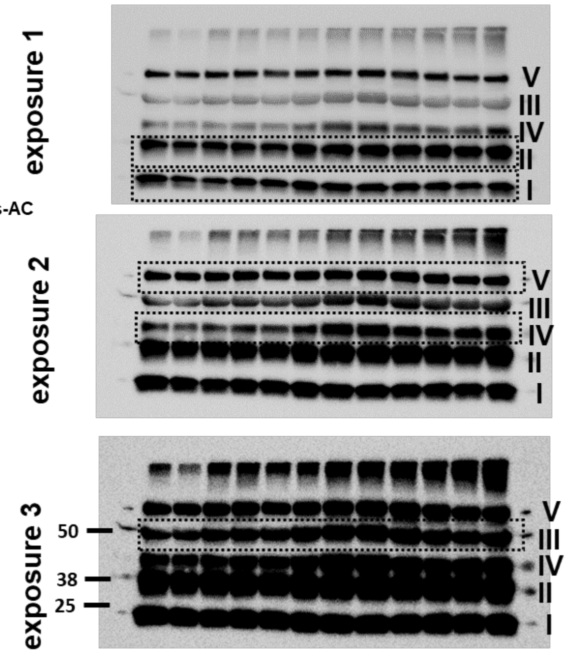

S3d

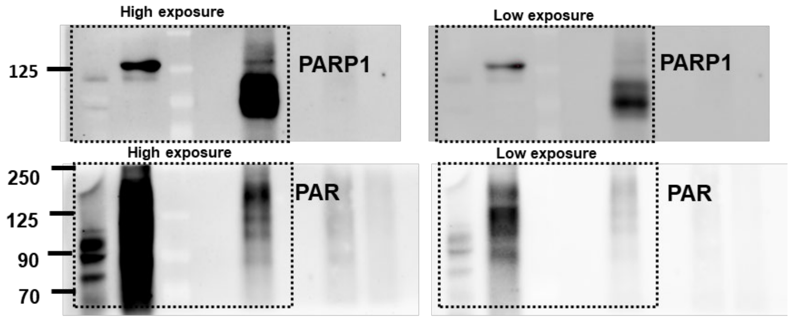

S4a

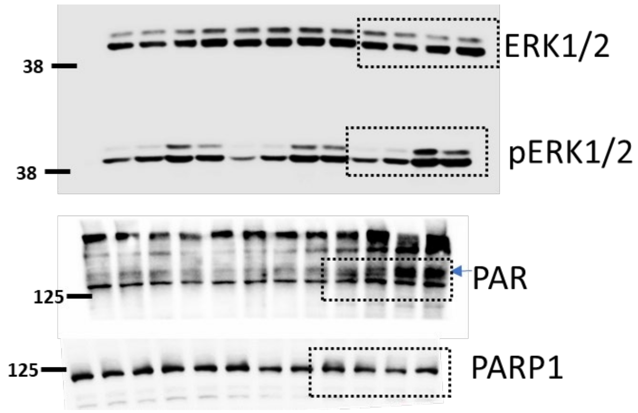

S4b

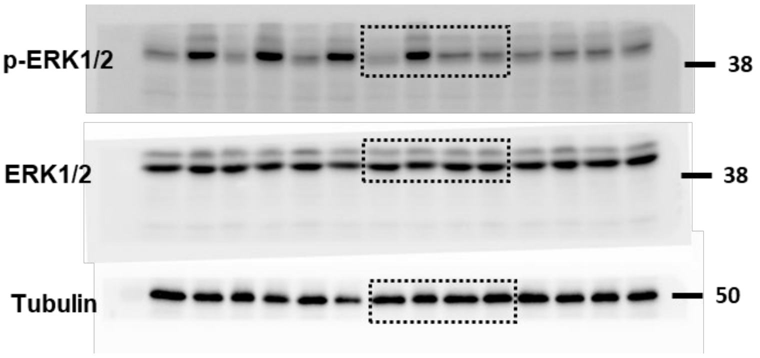

S4c

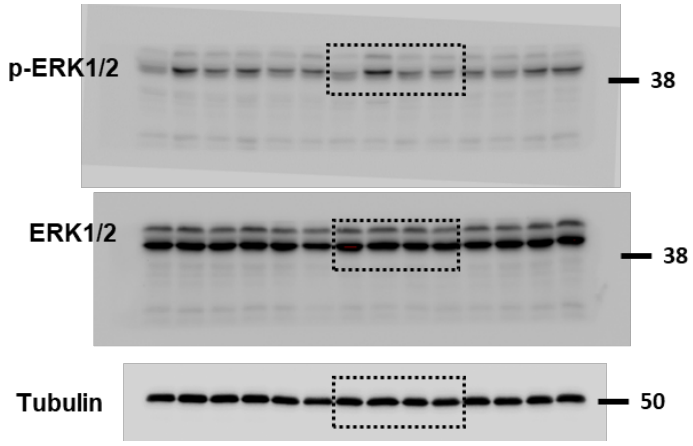

S4d

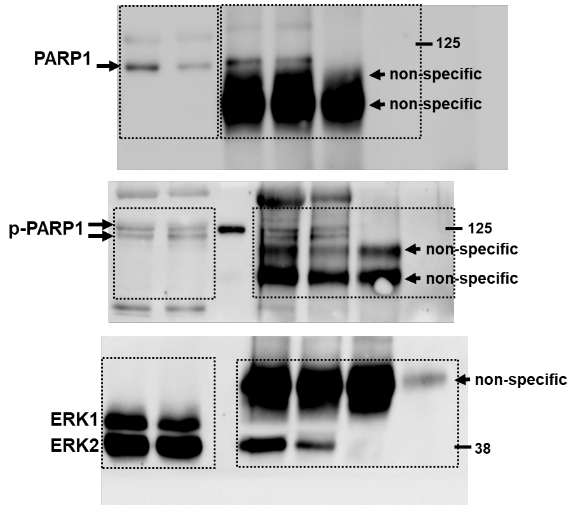
PARP1
125
non-specific
non-specific
p-PARP1
125
non-specific
non-specific
ERK1
ERK2
non-specific
38

S4e

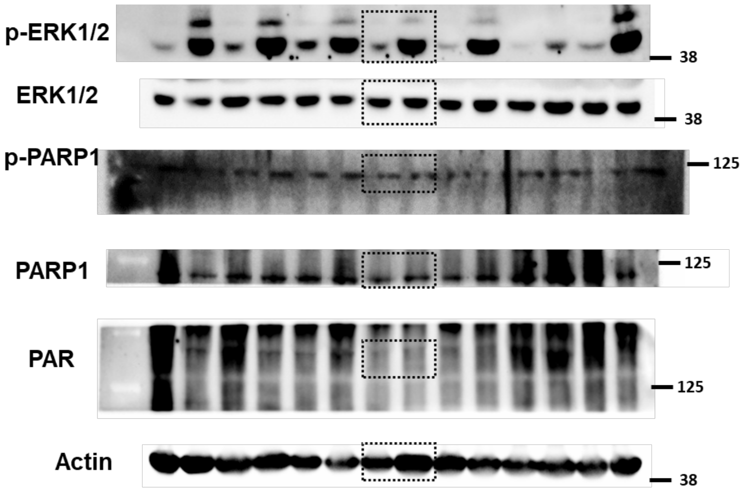
p-ERK1/2
38
ERK1/2
38
p-PARP1
125
PARP1
125
PAR
125
Actin
38

S5a

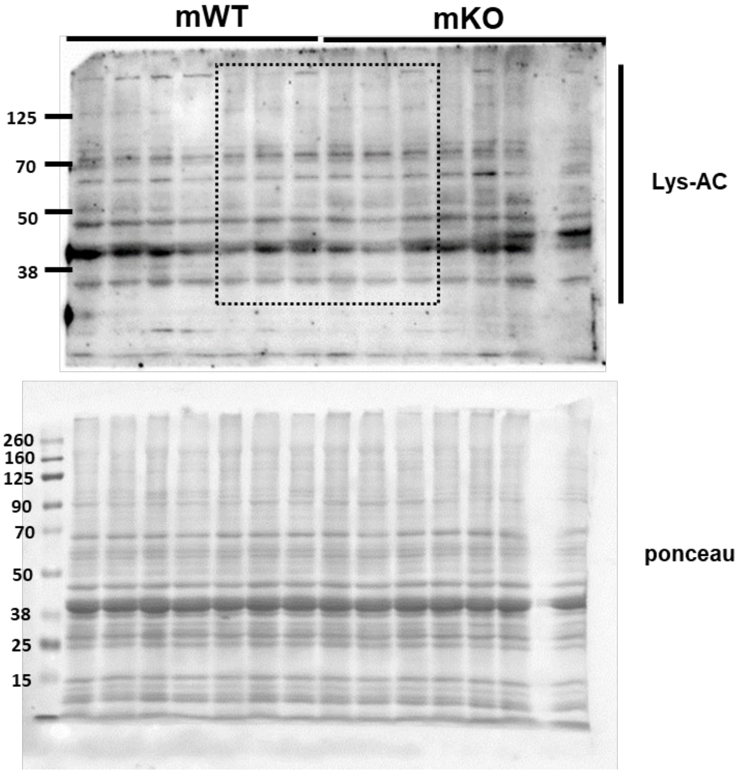
mWT
mKO
125
70
50
38
Lys-AC
260
160
125
90
70
50
38
25
15
ponceau

Supplementary tables

Supplementarytable 1. Key reagents			
Technique	Reagent	Vendor	Catalog
Mouse Genotyping	NaOH	Sigma-Aldrich	221465-2.5KG
	Tris base	ThermoFisher	BP152-5
	ChoiceTaq	Thomas Scientific	C775Y45
	TaqReadyMix	Sigma-Aldrich	P4600-100RXN
	PlatinumTaq	ThermoFisher	10966034
Mouse treatments	PJ-34	Cayman Chemicals	14440
	Pentobarbital	Sagent	676-20
	Sucralose	Sigma-Aldrich	69293-100G
	3OMG	Sigma-Aldrich	M4879-25G
	Aspartame	Cayman Chemicals	26089
Harvest and tissue processing	Pentobarbital	Sagent	676-20
	RNase-free plastic beads	Next Advance	GB05-RNA
	Sarstedt tubes	Sarstedt	72.694.406
Primary myocyte isolation and culture	PBS	Gibco	70011-044
	HBSS	Gibco	14175103
	Collagenase	Gibco	17101015
	Dispase II	Roche	4942078001
	DMEM	Sigma-Aldrich	D6429-500ml
	Fetal bovine serum	Gibco	16140-071
	Glutamax	Gibco	35050061
	Penicillin-streptomycin	Gibco	15140148
	Ham's F-10 nutrient mix	Gibco	11550043
	Basic fibroblast growth factor	Sigma-Aldrich	F0291-25UG
	Epithelial growth factor	Sigma-Aldrich	E9644
	Insulin	Sigma-Aldrich	I9278-5ML
	Dexamethasone	Sigma-Aldrich	D-4902
	Collagen	Gibco	A1048301
	Trypsin	Gibco	25200072
	Horse serum	Gibco	26050088
C2C12 culture and studies	Fetal bovine serum	Gibco	16140-071
	Pen/Strep	Gibco	15140148
	DMEM	Sigma-Aldrich	D6429-500ml
	DMEM, low glucose	Sigma-Aldrich	D6046-500ml
	Horse serum	Gibco	26050088
	Glucose	Gibco	A2494001
	3-OMG	Sigma-Aldrich	M4879-25G
	PJ-34	Cayman Chemicals	14440
	Gurmarin	MyBiosource	MBS407314
	Earle's Balanced Salt Solution	Sigma-Aldrich	E3024-6X500ML
	PD98059	Cayman Chemicals	10006726
	Epithelial growth factor	Sigma-Aldrich	E9644
	Sucralose	Sigma-Aldrich	69293-100G
	Phorbol 12-myristate 13-acetate (PMA)	Cayman Chemicals	10008014
Plasma glucose, insulin, and tolerance tests	Glucose	Sigma-Aldrich	G8270-5KG
	Insulin	Lily	HI-210
	Insulin ultrasensitive ELISA	ChrystalChem	90080
RNA isolation, gene expression, and analysis	TRIzol	ThermoFisher	15596018
	2.8-mm beads	Omni International	19-646-3
	0.05-mm glass beads	Next Advance	GB05-RNA
	Chloroform	Sigma-Aldrich	C2432-500ML
	1.5-mL tubes	Axygen	MCT-150-C-S
	Ethanol	Fisher	BP2818
	Direct-zol RNA Microprep kit	Zymo Research	R2062
	High-Capacity cDNA Reverse Transcription kit	ThermoFisher	4368814

Supplementary table 1 (continued). Key reagents			
Ribotag RNA isolation	PBS	Gibco	70011-044
	Cycloheximide	Sigma-Aldrich	239764-100MG
	Tris pH 7.4	Invitrogen	15568-025
	MgCl	Invitrogen	AM9530G
	KCl	Invitrogen	AM9640G
	DTT	Sigma-Aldrich	D9779-25G
	Triton X-100	Sigma-Aldrich	93443-100ML
	Cycloheximide	Sigma-Aldrich	239764-100MG
	Dounce Homogenizer	Wilmad Labglass	LG10645090
	AG Magnetic Beads	ThermoFisher	88803
	TRIzol	ThermoFisher	15596018
	Direct-zol RNA Microprep kit	Zymo Research	R2062
Histochemistry	Optimal cutting temperature (OCT) compound	Fisher	23-730-571
	PBS	Gibco	70011-044
	Sucrose	VWR	97061-432
	Prolong Gold Antifade Mountant	ThermoFisher	P36930
Electron microscopy	Glutaraldehyde	Sigma-Aldrich	G6257-10X10ML
	Potassium phosphate dibasic	JT Baker	3250-01
	Potassium phosphate monobasic	Sigma-Aldrich	P9791-500G
	Ethanol	Fisher	BP2818
	Eponate 12 epoxy resin	Ted Pella, Inc.	18012
NAD cycling assay	Perchloric acid	Sigma-Aldrich	311421-50ML
	PBS	Gibco	70011-044
	BSA	Pierce	23209
	Nicotinamide	Sigma-Aldrich	72345-50G
	Ethanol	ThermoFisher	BP2818500
	FMN	Sigma-Aldrich	F6750-5G
	Resazurin	Cayman Chemicals	14322
	ADH	Sigma-Aldrich	A3263-30KU
	Diaphorase	Sigma-Aldrich	D5540-100UN
	NaOH	Sigma-Aldrich	221465-2.5KG
	BCA kit	ThermoFisher	PI23225
Protein extraction	PVDF membrane	Immobilon	IPFL00010
	Nitrocellulose membrane	ThermoFisher	10600004
	RIPA buffer	ThermoFisher	89900
	cOmplete Mini Protease Inhibitor Cocktail	Roche	04693124001
	PhosStop Phosphatase Inhibitor Cocktail Tablets	Roche	04906837001
	SDS 6X Reducing buffer	ThermoFisher	AAJ61337AD
Immunoblotting	Acrylamide	Bio-Rad	1610158
	PVDF membrane	Immobilon	IPFL00010
	Nitrocellulose membrane	ThermoFisher	10600004
	Ponceau	Sigma-Aldrich	P7170-1L
	Dehydrated milk	Bio-Rad	1706404
	SuperSignal reagent	ThermoFisher	37074
Nuclear isolation	PBS	Gibco	70011-044
	SIGMAFAST Protease Inhibitor Cocktail Tablets, EDTA-Free	Sigma	S8830-20TAB
	PhosStop Phosphatase Inhibitor Cocktail Tablets	Roche	04 906 837 001
	Dounce homogenizer	Wilmad Labglass	LG10645090
	NE-PER Nuc Cyt Kit	ThermoFisher	78833
	CER I	ThermoFisher	78833
	CER II	ThermoFisher	78833
	NER	ThermoFisher	78833
Immuno-precipitation	RIPA buffer	ThermoFisher	89900
	SIGMAFAST Protease Inhibitor Cocktail Tablets, EDTA-Free	Sigma	S8830-20TAB
	PhosStop Phosphatase Inhibitor Cocktail Tablets	Roche	04 906 837 001
	Protein A/G-conjugated beads	ThermoFisher	88803
	SDS 6X reducing buffer	ThermoFisher	AAJ61337AD

Supplementarytable 2. qPCR primers		
Target (geneID)	Fw	Rv
18s	GTAACCCGTTGAACCCCATT	CCA TCC AAT CGG TAG TAG CG
tas1r2	GAACTGCCCACCAACTACAA	CCA TCG TGG ACA GAC ATG AA
tas1r3	CCAGTGAGTCTTGGCTGACA	TTC AGT GAG GCA CAG AAT GC
Cre (Myogenin-cre)	CCGGTGAACGTGCAAAACAGGCTCTA	CTTCCAGGGCGCGAGTTGATAGC
icre (Tas1r2-Cre)	AGATGCCAGGACATCAGGAACCTG	ATC AGC CAC ACC AGA CAC AGA GAT C

Supplementarytable 3. Genotyping primers and protocols				
Allele	Primer	Sequence	Amplicon size	Polymerase
Cre (Myo-Cre)	CRE Fw	CCGGTGAACGTGCAAAACAGGCTCTA	Cre Mutant: 400 bp	ChoiceTaq
	CRE Rv	CTTCCAGGGCGCGAGTTGATAGC	-	
	Genome Fw	CTAGGCCACAGAATTGAAAGATCT	Genome: 324 bp	
	Genome Rv	GTAGGTGGAAATTCTAGCATCATCC	-	
RiboTag	Ribo Fw	GGGAGGCTTGCTGGATATG	WT: 243bp	ChoiceTaq
	Ribo Rv	TTTCCAGACACAGGCTAAGTACAC	Ribo/Ribo: 290bp	
Flox	T1R2 Flox Fw	TGATAGGGAAGAGGGAGAAGGAGGG	fl/fl: 712 bp	PlatinumTaq
	T1R2 Flox Rv	TCACTAATACCATCACCAGCACCC	WT: 542 bp	
hT1R2	hT1R2 Mut Fw	AGGTGAACTTCAAGATCCGCC ACA	Mutant: 909 bp	ChoiceTaq
	hT1R2 Com Rv	TGCTTACATAGTCTAACTCGCGAC	WT: 580 bp	
	hT1R2 WT Fw	AGCACTTGCTCTCCCAAAGTC	-	
T1R2-iCRE	iCre Fw	AGATGCCAGGACATCAGGAACCTG	Mutant: 236 bp	ChoiceTaq
	iCre Rv	ATCAGCCACACCAGACACAGAGATC	-	
Tomato	Tomato WT Fw	AAGGGAGCTGCAGTGGAGTA	WT: 297 bp	Choice Taq
	Tomato WT Rv	CCGAAAATCTGTGGGAAGTC	-	
	Tomato Mut Fw	CTGTTCCTGTACGGCATGG	Mutant: 200 bp	
	Tomato Mut Rv	GGCATTAAAGCAGCGTATCC	-	

Suplementarytable 4. Antibody information							
Application	Target protein	Target epitope	Vendor	Clonality	Source	Catalog number	Dilution
Western blot	Actin	C-terminus	Sigma-Aldrich	Monoclonal AC-40	Mouse	A3853	1:1000
Western blot	Actinin	Total	Cell Signaling Technology	Monoclonal D6F6	Rabbit	6487S	1:1000
Western blot	Tubulin	Total	Sigma-Aldrich	Monoclonal DM1A	Mouse	T6199	1:1000
Western blot	GAPDH	Total	Cell Signaling Technology	Monoclonal 14C10	Rabbit	2118	1:1000
Western blot	Mitochondrial complexes	C1-C5 cocktail	Abcam	Polyclonal	Mouse	ab110413	1:1000
Western blot	akt	Total	Cell Signaling Technology	Monoclonal C67E7	Rabbit	4691	1:1000
Western blot	PAR	poly(ADP-ribose) chains	Enzo	Monoclonal 10H	Mouse	ALX-804-220-R100	1:500
Western blot	PARP1	Full-length (116kDa), large fragment (89 kDa)	Cell Signaling Technology	Polyclonal	Rabbit	9542L	1:1000
Western blot	NAMPT	Residue 400-450	Fortis Life Sciences	Polyclonal	Rabbit	A300-372A	1:1000
Western blot	PGC1a	Total (113 kDa and 38 kDa)	MilliporeSigma	Monoclonal 4C1.3	Mouse	ST1202	1:1000
Western blot	SIRT1	Residue 1-131	MilliporeSigma	Polyclonal	Rabbit	07-131	1:1000
Western blot	pAMPK	P-Thr172	Cell Signaling Technology	Monoclonal 40H9	Rabbit	2535	1:1000
Western blot	AMPK	Total	Cell Signaling Technology	Monoclonal D63G4	Rabbit	5832	1:1000
Western blot	LysAC	Acetylated lysine	Cell Signaling Technology	Polyclonal	Rabbit	9441	1:1000
Western blot	P-ERK 1/2	ERK1 P-Thr202, ERK1 P-Thr202/P-Tyr204 , ERK2 P-Thr185/Tyr187	Cell Signaling Technology	Monoclonal D13.14.4E	Rabbit	4370	1:1000
Western blot	ERK 1/2	Total	Cell Signaling Technology	Monoclonal 137F5	Rabbit	4695	1:1000
Western blot	pPARP	P-S372	NSJ Bioreagents	Polyclonal	Rabbit	F48728	1:1000
Western blot	WB ms secondary IgG	Total mouse IgG	Cell Signaling Technology	Polyclonal	Horse	7076	1:3000
Western blot	WB rb secondary IgG	Total rabbit IgG	Cell Signaling Technology	Polyclonal	Goat	7074	1:3000
Immunoprecipitation	antiHA	HA tag	Abcam	Polyclonal	Rabbit	ab9110	1:200
Immunoprecipitation	PARP1	Full-length (116kDa), large fragment (89 kDa)	Cell Signaling Technology	Polyclonal	Rabbit	9542L	1:125
