# Supplementary material for: The TAS1R2 G-protein-coupled receptor is an ambient glucose sensor in skeletal muscle that regulates NAD homeostasis and mitochondrial capacity
Source: Nat Commun. 2024 Jun 8;15:4915. doi: 10.1038/s41467-024-49100-8 (PMC11162498; doi:10.1038/s41467-024-49100-8)
Supplement: Supplementary file 3 — Reporting Summary [file 41467_2024_49100_MOESM3_ESM.pdf]

nature portfolio

Corresponding author(s): GEORGE A KYRIAZIS

Last updated by author(s): Feb 8, 2023

Reporting Summary

Nature Portfolio wishes to improve the reproducibility of the work that we publish. This form provides structure for consistency and transparency in reporting. For further information on Nature Portfolio policies, see our Editorial Policies and the Editorial Policy Checklist.

Statistics

For all statistical analyses, confirm that the following items are present in the figure legend, table legend, main text, or Methods section.

n/a	Confirmed	
☐	☒	The exact sample size (*n*) for each experimental group/condition, given as a discrete number and unit of measurement
☐	☒	A statement on whether measurements were taken from distinct samples or whether the same sample was measured repeatedly
☐	☒	The statistical test(s) used AND whether they are one- or two-sided<br>*Only common tests should be described solely by name; describe more complex techniques in the Methods section.*
☐	☒	A description of all covariates tested
☐	☒	A description of any assumptions or corrections, such as tests of normality and adjustment for multiple comparisons
☐	☒	A full description of the statistical parameters including central tendency (e.g. means) or other basic estimates (e.g. regression coefficient) AND variation (e.g. standard deviation) or associated estimates of uncertainty (e.g. confidence intervals)
☐	☒	For null hypothesis testing, the test statistic (e.g. *F*, *t*, *r*) with confidence intervals, effect sizes, degrees of freedom and *P* value noted<br>*Give P values as exact values whenever suitable.*
☒	☐	For Bayesian analysis, information on the choice of priors and Markov chain Monte Carlo settings
☒	☐	For hierarchical and complex designs, identification of the appropriate level for tests and full reporting of outcomes
☒	☐	Estimates of effect sizes (e.g. Cohen's *d*, Pearson's *r*), indicating how they were calculated

Our web collection on statistics for biologists contains articles on many of the points above.

Software and code

Policy information about availability of computer code

Data collection	TSE Phenomaster V 7.6.2.10970 (TSE Systems) for food and water intake mouse<br>EcoMRI (EchoMRI LLC) for body composition mouse<br>Oxygraph 2K with Datlab 5 software (Oroboros Instruments, Innsbruck, Austria) for ex vivo muscle respiration<br>Zeiss LSM 900 confocal (Zeiss) for imaging<br>Exer 6 treadmill (Columbus Instruments) for running mouse<br>FEI Technai G2 Spirit transmission electron microscope (Thermo Fisher Scientific) for imaging
Data analysis	The jamovi project (2021). jamovi. (Version 2.2.2.5.0) for statistics<br>GraphPad Prism 9.5.0.730 for statistics<br>Microsoft® Excel® for Microsoft 365 MSO (Version 2210 Build 16.0.15726.20188) 64-bit for statistics<br>Biorender (Science Suite, Inc.)

For manuscripts utilizing custom algorithms or software that are central to the research but not yet described in published literature, software must be made available to editors and reviewers. We strongly encourage code deposition in a community repository (e.g. GitHub). See the Nature Portfolio guidelines for submitting code & software for further information.

Data

Policy information about availability of data

All manuscripts must include a data availability statement. This statement should provide the following information, where applicable:

- Accession codes, unique identifiers, or web links for publicly available datasets
- A description of any restrictions on data availability
- For clinical datasets or third party data, please ensure that the statement adheres to our policy

The data generated and analyzed for the current study are available from the corresponding author (GAK). The source data generated in this study are provided in the Source Data file.

Human research participants

Policy information about studies involving human research participants and Sex and Gender in Research.

Reporting on sex and gender	n/a
Population characteristics	n/a
Recruitment	n/a
Ethics oversight	n/a

Note that full information on the approval of the study protocol must also be provided in the manuscript.

Field-specific reporting

Please select the one below that is the best fit for your research. If you are not sure, read the appropriate sections before making your selection.

☒ Life sciences ☐ Behavioural & social sciences ☐ Ecological, evolutionary & environmental sciences

For a reference copy of the document with all sections, see nature.com/documents/nr-reporting-summary-flat.pdf

Life sciences study design

All studies must disclose on these points even when the disclosure is negative.

Sample size	Male mice from each genotype (n=5-22) depending on the measured outcome.
Data exclusions	n/a
Replication	In vivo experiments were performed in at least 2 independent cohorts. Ex vivo and in vitro experiments were performed at least 3 times independently.
Randomization	Mice from each genotype were randomly assigned to perspective cohorts
Blinding	Data collection was performed using mouse or samples IDs.

Reporting for specific materials, systems and methods

We require information from authors about some types of materials, experimental systems and methods used in many studies. Here, indicate whether each material, system or method listed is relevant to your study. If you are not sure if a list item applies to your research, read the appropriate section before selecting a response.

Materials & experimental systems

n/a	Involved in the study	
☐	☒	Antibodies
☐	☒	Eukaryotic cell lines
☒	☐	Palaeontology and archaeology
☐	☒	Animals and other organisms
☒	☐	Clinical data
☒	☐	Dual use research of concern

Methods

n/a	Involved in the study	
☒	☐	ChIP-seq
☒	☐	Flow cytometry
☒	☐	MRI-based neuroimaging

Antibodies

Antibodies used

Western blot Actin C-terminus Sigma-Aldrich Monoclonal AC-40 Mouse A3853 Mouse, human 1:1000 Milk
Western blot Actinin Total Cell Signaling Technology Monoclonal D6F6 Rabbit 6487S Human, mouse, rabbit, monkey 1:1000 Milk
Western blot Tubulin Total Sigma-Aldrich Monoclonal DM1A Mouse T6199 Yeast, mouse, amphibian, human, rat, chicken, fungi, bovine 1:1000 Milk
Western blot GAPDH Total Cell Signaling Technology Monoclonal 14C10 Rabbit 2118 Human, mouse, rabbit, monkey, bovine, pig 1:1000 Milk
Western blot Mitochondrial complexes C1-C5 cocktail Abcam Polyclonal Mouse ab110413 Mouse, rat, cow, human, cynomolgus monkey 1:1000 Milk
Western blot akt Total Cell Signaling Technology Monoclonal C67E7 Rabbit 4691 Human, Mouse, Rat, Monkey, Drosophila 1:1000 Milk
Western blot PAR poly(ADP-ribose) chains Enzo Monoclonal 10H Mouse ALX-804-220-R100 Human, mouse, rat, Drosophila 1:500 Milk
Western blot PARP1 Full-length (116kDa), large fragment (89 kDa) Cell Signaling Technology Polyclonal Rabbit 9542L Human, mouse, rat, monkey 1:1000 Milk
Western blot NAMPT Residue 400-450 Fortis Life Sciences Polyclonal Rabbit A300-372A Human, mouse 1:1000 Milk
Western blot PGC1a Total (113 kDa and 38 kDa) MilliporeSigma Monoclonal 4C1.3 Mouse ST1202 Mouse, human, rat 1:1000 Milk
Western blot SIRT1 Residue 1-131 MilliporeSigma Polyclonal Rabbit 07-131 Human, mouse 1:1000 Milk
Western blot LysAC Acetylated lysine Cell Signaling Technology Polyclonal Rabbit 9441 All 1:1000 Milk
Western blot P-ERK ERK1 P-Thr202, ERK1 P-Thr202/P-Tyr204 , ERK2 P-Thr185/Tyr187 Cell Signaling Technology Monoclonal D13.14.4E Rabbit 4370 Human, mouse, rat, hamster, monkey, mink, Drosophila, zebrafish, bovine, dog, pig, S. cerevisiae 1:1000 Milk
Western blot ERK Total Cell Signaling Technology Monoclonal 137F5 Rabbit 4695 Human, mouse, rabbit, hamster, monkey, mink, Drosophila, zebrafish, bovine, dog, pig, C. elegans 1:1000 Milk
Western blot pPARP P-S372 NSJ Bioreagents Polyclonal Rabbit F48728 Human 1:1000 Milk
Western blot WB ms secondary IgG Total mouse IgG Cell Signaling Polyclonal Horse 7076 Mouse 1:3000 Milk
Western blot WB rb secondary IgG Total rabbit IgG Cell Signaling Polyclonal Goat 7074 Rabbit 1:3000 Milk
Immunoprecipitation antiHA HA tag Abcam Polyclonal Rabbit ab9110 Species-independent 1:200 Polysome Buffer
Immunoprecipitation PARP1 Full-length (116kDa), large fragment (89 kDa) Cell Signaling Technology Polyclonal Rabbit 9542L Human, mouse, rat, monkey 1:125 RIPA

Validation

Anti-mitochondrial has been validated by Abcam (https://www.abcam.com/total-oxphos-rodent-wb-antibody-cocktail-ab110413.html)
Anti-AKT has been validated by Cell Signaling Technology (https://www.cellsignal.com/products/primary-antibodies/akt-pan-c67e7-rabbit-mab/4691).
Anti-PAR has been validated in several previous works (https://www.enzolifesciences.com/ALX-804-220/poly-adp-ribose-monoclonal-antibody-10h/).
Anti-PARP1 has been validated by Cell Signaling Technology and in several previous works (https://www.cellsignal.com/products/primary-antibodies/akt-pan-c67e7-rabbit-mab/4691).
Anti-NAMPT has been validated by Fortis Life Sciences and in several previous works (https://www.fortislife.com/products/primary-antibodies/rabbit-anti-pbef-antibody/BETHYL-A300-372).
Anti-PGC1a has been validated by MilliporeSigma and in several previous works (https://www.sigmaaldrich.com/US/en/product/mm/st1202).
Anti-SIRT1 has been validated by MilliporeSigma and in several previous works (https://www.sigmaaldrich.com/US/en/product/mm/07131).
Anti-LysAC has been validated by Cell Signaling Technology and in several previous works (https://www.cellsignal.com/products/primary-antibodies/acetylated-lysine-antibody/9441).
Anti-P-ERK has been validated by Cell Signaling Technology and in several previous works (https://www.cellsignal.com/products/primary-antibodies/phospho-p44-42-mapk-erk1-2-thr202-tyr204-d13-14-4e-xp-rabbit-mab/4370).
Anti-ERK has been validated by Cell Signaling Technology and in several previous works (https://www.cellsignal.com/products/primary-antibodies/p44-42-mapk-erk1-2-137f5-rabbit-mab/4695).
Anti-mouse IgG has been validated in several previous works (https://www.cellsignal.com/products/secondary-antibodies/anti-mouse-igg-hrp-linked-antibody/7076)
Anti-rabbit IgG has been validated in several previous works (https://www.cellsignal.com/products/secondary-antibodies/anti-rabbit-igg-hrp-linked-antibody/7074)
Anti-HA has been validated by Abcam and in several previous works (https://www.abcam.com/ha-tag-antibody-chip-grade-

ab9110.html).
Anti-PARP1 has been validated by Cell Signaling Technology and in several previous works (https://www.cellsignal.com/products/primary-antibodies/akt-pan-c67e7-rabbit-mab/4691).

Eukaryotic cell lines

Policy information about cell lines and Sex and Gender in Research

Cell line source(s)	C2C12 (ATCC CRL-1772) murine myocytes was a gift by Dr. Baskin. Primary murine myocytes were isolated as described in the methods.
Authentication	We differentiated the C2C12 cell line and primary myocytes and confirmed formation of contractile myotubes and the production of muscle proteins.
Mycoplasma contamination	All cell lines tested negative for mycoplasma.
Commonly misidentified lines (See ICLAC register)	N/A

Animals and other research organisms

Policy information about studies involving animals; ARRIVE guidelines recommended for reporting animal research, and Sex and Gender in Research

Laboratory animals

All animal procedures were performed under the approval of The Ohio State University institutional animal care and use committee (IACUC). Mice were housed on a 12 h light/dark cycle with free access to water and standard diet. Male mice were used for experimental purposes only after reaching sexual maturity at 12-16 weeks of age unless otherwise stated.
Tas1r2 floxed mice (Tas1r2fl/fl) were generated from the KOMP Repository (#CSD25803). Tas1r2 iCre knock in mice (Tas1r2iCre) were previously generated as described1. Human TAS1R2 transgenic mice (hTAS1R2fl-stop) were created by Ingenious targeting lab (NY) by placing a cDNA cassette containing a synthetic CAG promoter, a floxed NEO stop cassette, the consensus human TAS1R2 cDNA, an IRES-EGFP and a BGH polyA on the mouse ROSA26 locus. Whole body Tas1r2 knockout mice (bKO) were a gift from Dr. Zuker 2. Myogenin-Cre mice (MyoCre) were a gift by Dr. Olson 3. The above mouse lines (hTAS1R2fl-stop, Tas1r2fl/fl, Tas1r2iCre, and MyoCre) were backcrossed and then maintained to pure C57BL6/6J strain (using Jax# 000664). RiboTag-Rpl22fl/fl-HA (RiboTagfl/fl) (Jax# 029977), tdTomato mice (Tdfl/fl) (Jax#007914) were obtained from the Jackson Lab.
Tdfl/fl mice were crossed with Tas1r2iCre mice to generate mice (TdTas1r2) that express Tdtomato in Tas1r2 positive cells1.
RiboTagfl/fl mice were crossed with MyoCre mice to generate mice (HA-RiboTagMyo) that express Rpl22-HA in myogenin positive cells (myocytes).
Tas1r2fl/fl mice were crossed with MyoCre mice to generate mice (mKO) with muscle-specific deletion of Tas1r2. mKO mice were crossed with hTAS1R2fl-stop mice to generate mice (mTg) with muscle-specific transgenic overexpression of human TAS1R2 without endogenous Tas1r2. We used a specific breeding scheme to obtain wild type control (mWT), mKO, and mTg congenic littermates: Congenic male mice carrying one MyoCre allele, one hTAS1R2fl-stop allele and two Tas1r2fl/fl alleles were crossed with congenic female mice carrying two Tas1r2fl/fl alleles. Male offspring carrying two Tas1r2fl/fl alleles were used as wild-type controls (mWT). Male offspring carrying one MyoCre allele and two (floxed out) Tas1r2fl/fl alleles were used for muscle-specific genetic deletion of Tas1r2 gene (mKO). Male offspring carrying one MyoCre allele, two (floxed out) Tas1r2fl/fl alleles, and one (floxed STOP) hTAS1R2fl-stop allele were used as muscle-specific transgenic expression of hTAS1R2 gene without the endogenous mouse Tas1r2 gene (mTg).

Wild animals	N/A
Reporting on sex	Male mice were used and reported.
Field-collected samples	N/A
Ethics oversight	All animal procedures were performed under the approval of The Ohio State University institutional animal care and use committee (IACUC).

Note that full information on the approval of the study protocol must also be provided in the manuscript.
