# Supplementary material for: The TAS1R2 G-protein-coupled receptor is an ambient glucose sensor in skeletal muscle that regulates NAD homeostasis and mitochondrial capacity
Source: Nat Commun. 2024 Jun 8;15:4915. doi: 10.1038/s41467-024-49100-8 (PMC11162498; doi:10.1038/s41467-024-49100-8)
Supplement: Supplementary file 4 — Source data [file 41467_2024_49100_MOESM4_ESM.docx]

**Inventory of supplementary information for The TAS1R2 G-protein-coupled receptor is an ambient glucose sensor in skeletal muscle that regulates NAD homeostasis and mitochondrial capacity**

**Supplementary figures 1-5**

Supplementary Fig. 1 | The Tas1r2 and Tas1r3 sweet taste GPCRs are expressed in skeletal muscle fibers.

Supplementary Fig. 2 | GPCRs expression in skeletal myofibers (myocytes).

Supplementary Fig. 3 | TAS1R2-mediated glucose sensing regulates NAD levels coupled to PARP activity.

Supplementary Fig. 4 | TAS1R2 activates the ERK2-PARP1 axis in skeletal muscle.

Supplementary Fig. 5 | TAS1R2 deficiency in skeletal muscle does not affect growth and glucose homeostasis.

**Western blot scans**

**Supplementary tables 1-4**

Supplementary Table 1 | Key reagents.

Supplementary Table 2 | qPCR primers.

Supplementary Table 3 | Genotyping primers and protocols.

Supplementary Table 4 | Antibody information.
